# Pyrosequencing-Based Assessment of the *Bacteria* Diversity in Surface and Subsurface Peat Layers of a Northern Wetland, with Focus on Poorly Studied Phyla and Candidate Divisions

**DOI:** 10.1371/journal.pone.0063994

**Published:** 2013-05-21

**Authors:** Yulia M. Serkebaeva, Yongkyu Kim, Werner Liesack, Svetlana N. Dedysh

**Affiliations:** 1 Winogradsky Institute of Microbiology, Russian Academy of Sciences, Moscow, Russia; 2 Max Planck Institute for Terrestrial Microbiology, Marburg, Germany; University of Waterloo, Canada

## Abstract

Northern peatlands play a key role in the global carbon and water budget, but the bacterial diversity in these ecosystems remains poorly described. Here, we compared the bacterial community composition in the surface (0–5 cm depth) and subsurface (45–50 cm) peat layers of an acidic (pH 4.0) *Sphagnum*-dominated wetland, using pyrosequencing of 16****S rRNA genes. The denoised sequences (37,229 reads, average length ∼430 bp) were affiliated with 27 bacterial phyla and corresponded to 1,269 operational taxonomic units (OTUs) determined at 97% sequence identity. Abundant OTUs were affiliated with the *Acidobacteria* (35.5±2.4% and 39.2±1.2% of all classified sequences in surface and subsurface peat, respectively), *Alphaproteobacteria* (15.9±1.7% and 25.8±1.4%), *Actinobacteria* (9.5±2.0% and 10.7±0.5%), *Verrucomicrobia* (8.5±1.4% and 0.6±0.2%), *Planctomycetes* (5.8±0.4% and 9.7±0.6%), *Deltaproteobacteria* (7.1±0.4% and 4.4%±0.3%), and *Gammaproteobacteria* (6.6±0.4% and 2.1±0.1%). The taxonomic patterns of the abundant OTUs were uniform across all the subsamples taken from each peat layer. In contrast, the taxonomic patterns of rare OTUs were different from those of the abundant OTUs and varied greatly among subsamples, in both surface and subsurface peat. In addition to the bacterial taxa listed above, rare OTUs represented the following groups: *Armatimonadetes*, *Bacteroidetes*, *Chlamydia*, *Chloroflexi*, *Cyanobacteria*, *Elusimicrobia*, *Fibrobacteres*, *Firmicutes*, *Gemmatimonadetes*, *Spirochaetes*, AD3, WS1, WS4, WS5, WYO, OD1, OP3, BRC1, TM6, TM7, WPS-2, and FCPU426. OTU richness was notably higher in the surface layer (882 OTUs) than in the anoxic subsurface peat (483 OTUs), with only 96 OTUs common to both data sets. Most members of poorly studied phyla, such as the *Acidobacteria*, *Verrucomicrobia*, *Planctomycetes* and the candidate division TM6, showed a clear preference for growth in either oxic or anoxic conditions. Apparently, the bacterial communities in surface and subsurface layers of northern peatlands are highly diverse and taxonomically distinct, reflecting the different abiotic conditions in microhabitats within the peat profile.

## Introduction

Northern wetlands are water-saturated, peat-accumulating ecosystems located between 45 and 70°N. These environments are recognized as a persistent sink for atmospheric CO_2_, with carbon accumulation rates of 10–30 g C m^−2^ year^−1^. They harbor a total of 200–450 Pg of carbon, which represents about one-third of the global soil carbon pool [1]. Northern wetlands are also known as a major source of the greenhouse gas methane (CH_4_), which is produced in the anoxic peat layers due to decomposition of organic matter [2]–[4]. In addition to their importance as a large terrestrial carbon store, these wetlands hold a key role in the global water balance and represent one of the largest reservoirs of freshwater in the Northern Hemisphere. A large proportion of northern wetlands consists of *Sphagnum*-dominated bogs, which occupy about 3% of the Earth’s terrestrial surface [5], comprising up to 80% of the area in some regions of West Siberia. Bogs are ombrotrophic ecosystems that are decoupled from the groundwater of the surrounding watershed and receive water and nutrients only via atmospheric deposition. These wetlands are highly acidic (pH values typically below 4.0) and nutrient-poor by nature. Peat water usually contains very low concentrations of mineral N, S, and Fe and, therefore, redox transformations of these elements are only of minor importance in ombrotrophic bogs. Degradation of *Sphagnum*-derived litter is the basis of the microbial food web in these ecosystems (reviewed in [6]).

Despite the global importance of *Sphagnum*-dominated wetlands as both terrestrial carbon store and freshwater reservoir, the microbial diversity in these ecosystems remains largely unexplored [6]. The research to date has primarily focused on several functional guilds such as methanotrophic bacteria [7]–[10], methanogenic archaea [11]–[14], sulfate reducers [15], cellulose degraders [16], and nitrogen-fixing microorganisms [17], [18]. A few studies assessed the total microbial diversity in acidic northern peatlands, using T-RFLP fingerprinting and/or clone library analysis as well as, more recently, pyrosequencing of 16****S rRNA genes [19]–[26]. These studies showed that ombrotrophic bogs are usually dominated by members of the poorly studied bacterial phyla, such as the *Acidobacteria*, *Planctomycetes*, and *Verrucomicrobia*, which are represented mostly by as-yet-uncultivated organisms with unknown physiologies and metabolic potentials. During the last decade, a number of peat-inhabiting acidobacteria and planctomycetes were obtained in pure culture and characterized (reviewed in [6]). These isolation-based studies provided the first proof for the presence of cellulose-degrading capabilities in some of the acidobacteria and planctomycetes [16], [27], [28] and highlighted the role of these bacteria as slow-acting decomposers in acidic and cold wetlands. With the exception of *Telmatobacter bradus* [27], however, all characterized peat-inhabiting acidobacteria and planctomycetes are aerobic bacteria, which can thrive only in the narrow surface zone of the peat bog profile. The occurrence of anammox planctomycetes in ombrotrophic peatlands has not yet been confirmed [29], although these bacteria were detected in a swampy peat soil fed by nitrate-enriched local groundwater [30]. There is even less known about the lifestyles of peat-inhabiting verrucomicrobia. To date, none of these bacteria has been cultivated from acidic peat. One intriguing question is whether the recently discovered acidophilic methanotrophic *Verrucomicrobia* [31] occur in acidic northern wetlands.

Here, we used 454-pyrosequencing to gain deeper insights into the bacterial diversity associated with surface (0–5 cm depth) and subsurface (45–50 cm) peat of an acidic (pH 4.0) *Sphagnum*-dominated ombrotrophic wetland. We hypothesized that oxygen availability is a major factor shaping bacterial community composition in *Sphagnum*-dominated peat bogs. We also aimed to test how subsampling affects the detectable diversity patterns. Our analyses were specifically focused on poorly understood and elusive groups of bacteria, such as *Acidobacteria*, *Verrucomicrobia*, *Planctomycetes*, and candidate division TM6.

## Materials and Methods

### Sampling Site

Acidic (pH 4.0) peat used in this study was sampled from the *Sphagnum*-dominated, ombrotrophic peat bog Obukhovskoye, Yaroslavl region, European North Russia (58° 14′N, 38° 12′E). Vegetation within this wetland is represented by *Sphagnum angustifolium*, *Sph. fuscum*, *Carex* spp., *Oxicoccus* sp. and *Vaccinium* sp. One set of four independent samples was collected from the surface peat layer (0–5 cm depth), while another set of four independent samples was taken from below the water table (located 7–10 cm beneath peat surface), at a depth of 45–50 cm. No specific permits were required for sampling at this wetland site. This location is not privately-owned or protected in any way and is also not part of a national park or reserve. Our sampling did not involve endangered or protected species. The samples were transported to the laboratory in boxes containing ice packs, homogenized by cutting the peat material into small fragments (about 0.5 cm) with sterile scissors, and frozen at −20°C for DNA extraction within 1 day after sampling.

### DNA Extraction, PCR Amplification and 454-pyrosequencing

Two sets of four subsamples (one each from surface and subsurface peat) were taken for DNA extraction. Each of the eight subsamples (0.5 g wet weight) was processed separately. The extraction of DNA was performed using a FastDNA SPIN kit for Soil (Bio101, Carlsbad, USA) according to the manufacturer’s instructions. Bacterial 16****S rRNA gene amplicons were generated using the universal primers 907F (5′-AAA CTY AAA KGA ATT GAC GG-3′) and 1392R (5′-ACG GGC GGT GTG TRC-3′) [32]. The 907F primer included a sample-specific 6-bp barcode. PCR amplification was performed in a DNA thermal cycler (model 9700; PE Applied Biosystems) under the following conditions: initial denaturation (3 min at 95°C); 30 cycles consisting of denaturation (30 sec at 95°C), primer annealing (45 sec at 55°C), and elongation (90 sec at 72°C), with a final elongation step for 8 min at 72°C. Quantification of the PCR products was performed using the Quant-iT dsDNA BR assay kit in combination with the Qubit fluorometer (Invitrogen GmbH, Karlsruhe, Germany). PCR products of each subsample were generated in triplicate, pooled in equal amounts and purified using the Wizard® SV Gel and PCR Clean-Up System (Promega, Madison, USA). The purified products were pyrosequenced at the Max Planck Genome Centre Cologne, Germany.

### Bioinformatic Analyses

The raw sequence data were processed and analyzed using the Quantitative Insights Into Microbial Ecology (QIIME v1.5.0) pipeline, with default settings [33]. Reads were removed from further analysis if at least one of the following criteria was met: (i) average quality score lower than 30, (ii) reads shorter than 200 bp, (iii) number of ambiguous bases greater than 6, and (iv) presence of homopolymers with more than 8 bp. The quality-filtered reads were denoised to remove sequencing errors by flowgram clustering. Clustering of the sequences into operational taxonomic units (OTUs) was performed using UCLUST [34], [35] and a cutoff value of 97% sequence identity. The most abundant sequence type within each OTU was selected to represent the respective OTU in further analysis. Taxonomic classification was performed using the RDP classifier and a confidence threshold of 80% [36]. Chao1 and Shannon indices were calculated to estimate taxon richness, diversity, and evenness. The community composition in surface and subsurface peat and between their subsamples was compared using weighted UniFrac distances in principal coordinate analysis (PCoA) [37]. OTU distribution between subsamples was analyzed, separately for surface and subsurface peat, by a custom-coded python script. Results were summarized in a table containing the information on taxonomic affiliation and the number of reads assigned to each OTU. Phylogenetic trees were constructed using the ARB program package [38]. For the calculation of putative sequencing error rates, multiple sequence alignments were generated in MOTHUR (v1.27 [39]) using SILVA reference database [40]. Sequences were visualized in MEGA v4 [41].

### Sequence Accession Numbers

The 454 pyrosequencing reads (raw data) have been deposited under the study number SRP016517 in the NCBI Sequence Read Archive, with the following accession numbers: SRP605326 (23,517 reads obtained from surface peat); and SRP600121 and SRP605331 (34,760 and 4,060 reads, respectively, obtained from subsurface peat).

## Results and Discussion

### Bacterial Taxon Richness and Diversity Coverage

Partial 16****S rRNA gene sequences were retrieved from an acidic *Sphagnum* peat of European North Russia. Quality filtration and denoising of the raw data resulted in a total of 42,096 sequences (average read length, ∼430 bp). Of these, 2,135 and 2,732 sequences were classified as belonging to members of the *Archaea* and *Eukarya*, respectively (tables S1 and S2), and were excluded from further analysis. The remaining 37,229 sequences were of bacterial origin. Of these, 697 sequences could not be assigned to any particular phylum (364 and 333 sequences from surface and subsurface peat, respectively). Nearly equal numbers of 454 reads were obtained for surface and subsurface peat. The subsamples I–IV from surface peat were represented by 4,314, 5,013, 4,574, and 5,009 bacterial sequences (total of 18,910 reads), while those from subsurface peat had 3,984, 6,778, 2,530, and 5,027 bacterial sequences (total of 18,319 reads).

**Figure 1:**
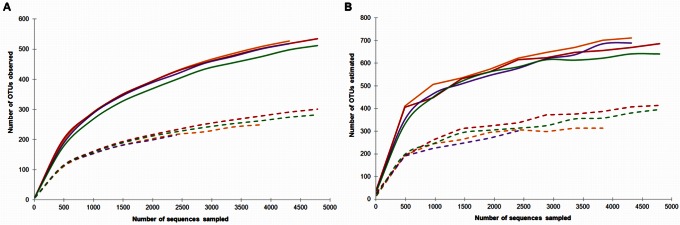
Bacterial taxon richness in acidic peat as assessed by barcoded pyrosequencing of 16 S rRNA genes. The rarefaction curves show the relation between the increase in bacterial OTUs and the number of randomly sampled sequences, separately for each subsample: I, orange; II, red; III, purple; and IV, green [experimentally observed richness (A) versus that predicted by Chao1 (B)]. The curves for the subsamples from surface and subsurface peat are shown by solid and dashed lines, respectively.

We used 97% sequence identity as the cutoff for cluster analysis at the species level [42]–[44]. Rarefaction curves constructed for the subsamples from surface peat exhibited a steeper slope than those determined for the subsamples from subsurface peat, demonstrating a greater bacterial richness in the oxic zone (Figure 1). Our pyrosequencing approach covered 74±4% of the species-level richness (Chao1) estimated for the subsamples from surface peat. The corresponding coverage values for subsurface peat ranged from 71±2% for subsample III (2,530 reads) to 74±4% for subsample IV (6,778 reads). Beta-diversity analysis revealed that the bacterial community composition in the subsamples from surface peat was highly similar to each other, but distinct to that in the subsamples from subsurface peat (Figure S1). Shannon`s *H* index was calculated to be 7.3±0.1 for the subsamples from surface peat, while the corresponding value for subsurface peat was 5.7±0.1 (Figure S2). Apparently, the bacterial community in the oxic surface layer had not only a greater species richness but also was more evenly structured than the community in the anoxic subsurface peat. Notably, random analysis of less than 1,000 sequences of each subsample was sufficient to observe a robust trend in the recovery of the abundant OTUs, in both surface and subsurface peat (Figure S2).

### Abundant versus Rare OTUs

The 16****S rRNA gene sequences retrieved from acidic *Sphagnum*-derived peat were affiliated with 27 bacterial phyla and corresponded to 1,269 species-level OTUs. The bacterial richness was notably higher in the oxic surface layer (882 OTUs) than in anoxic subsurface peat (483 OTUs), with only 96 OTUs common to both sequence data sets (10.9% and 19.9% of the bacterial OTUs identified in surface and subsurface peat, respectively).

**Figure 2:**
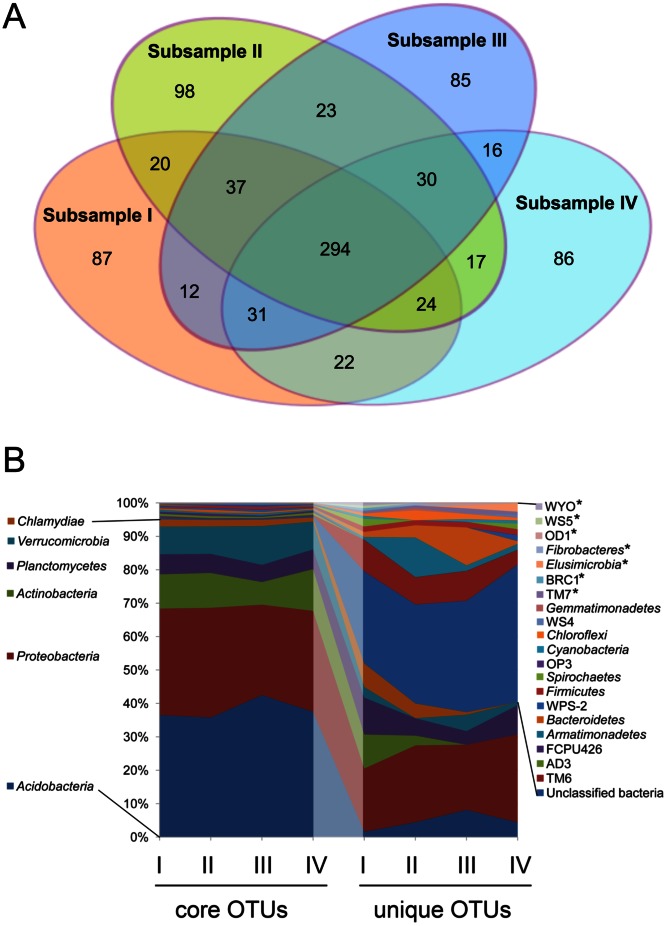
OTU distribution and relative sequence abundance of bacterial phyla and candidate divisions among the subsamples from surface peat. (A) Venn diagram showing the number of OTUs unique to a single subsample or shared by two, three, or all four subsamples. (B) Relative sequence abundance of bacterial phyla and candidate divisions based on OTUs that were detected either in all four subsamples (core OTUs) or only in a single subsample (unique OTUs). Phyla that were detected only in the surface layer, but not in subsurface peat, are marked by asterisks.

**Figure 3:**
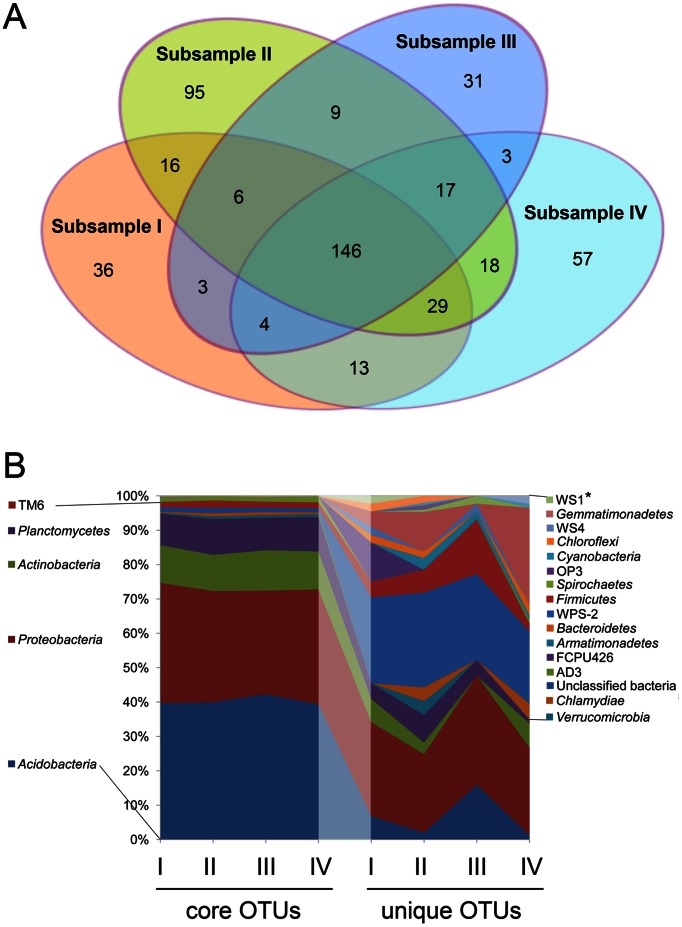
OTU distribution and relative sequence abundance of bacterial phyla and candidate divisions among the subsamples from subsurface peat. (A) Venn diagram showing the number of OTUs unique to a single subsample or shared by two, three, or all four subsamples. (B) Relative sequence abundance of bacterial phyla and candidate divisions based on OTUs that were detected either in all four subsamples (core OTUs) or only in a single subsample (unique OTUs). Phyla that were detected only in subsurface peat, but not in the surface layer, are marked by asterisks.

Abundant or core OTUs were compared with rare and unique OTUs. Core OTUs were detected in all four subsamples and, in the oxic surface layer, mostly affiliated with the *Acidobacteria*, *Proteobacteria*, *Actinobacteria*, *Verrucomicrobia*, *Planctomycetes*, and *Chlamydiae*, collectively termed core phyla. In the anoxic subsurface peat, *Acidobacteria*, *Proteobacteria*, *Actinobacteria*, *Planctomycetes*, and the candidate division TM6 represented the predominant core phyla. OTUs detected in two or three subsamples, but not all four subsamples, were defined as rare OTUs, while the subset of rare OTUs detected in only a single subsample of the respective peat layer was defined as unique OTUs (Figures 2 , 3 , S3).

The vast majority of the 356 (surface) and 219 (subsurface) unique OTUs were singletons (79.8% and 82.2%, respectively) and doubletons (10.7% and 8.2%, respectively). As low as 11 unique OTUs (1.9%) contained 6 to 13 reads. The 232 (surface layer) and 118 (subsurface peat) rare OTUs contained each between 2 and 20 reads, with the exception of one OTU that belonged to the acidobacterial subdivision 1. It was detected in only three subsamples of subsurface peat, but comprised 114 reads. Most of the OTUs detected in only two subsamples were composed of 2 to 5 reads, while the majority of those detected in three subsamples comprised 5–11 reads (Figure S4).

The relative sequence abundance of the core phyla was 95.2±0.3% (surface) and 95.3±0.3% (subsurface) among the OTUs detected in all four subsamples. The core phyla showed a uniform taxonomic pattern across the subsamples, in both surface and subsurface peat (Figures 2 , 3). Their relative abundance, however, declined to 74.1±3.6% (surface) and 85.3±2.9% (subsurface) among the rare OTUs to as low as 42.4±6.5% (surface) and 45.4±5.3% among the unique OTUs. As an opposite trend, the contribution of yet poorly characterized phyla and candidate divisions to the overall diversity patterns strongly increased from the core OTUs toward unique OTUs. The taxonomic patterns of unique OTUs varied greatly across subsamples and were clearly different from those of the core OTUs, for both surface (Figure 2) and subsurface peat (Figure 3).

Using one particular bacterial population, namely methanotrophs of the family *Methylococcaceae*, we were able to estimate the sequencing depth achieved in our study. A total of 100 16****S rRNA gene sequences of this family were retrieved from the oxic peat layer (20–30 reads from each of the four subsamples). The population size of these bacteria was assessed by fluorescence *in situ* hybridization with *Methylococcaceae*-specific probes M84+M705 and determined to be 1×10^5^ cells per gram of wet peat (Dedysh et al., unpublished data). Thus, we can roughly estimate that each unique OTU corresponds to a population size between 10^3^ and 10^5^ cells per gram of wet peat. Unique OTUs were affiliated with a wide range of bacterial phyla (*Armatimonadetes*, *Bacteroidetes*, *Chloroflexi*, *Cyanobacteria*, *Elusimicrobia*, *Fibrobacteres*, *Firmicutes*, *Gemmatimonadetes*, and *Spirochaetes*) and candidate divisions (AD3, WS1, WS4, WS5, WYO, OD1, OP3, BRC1, TM6, TM7, WPS-2, and FCPU426), in addition to those affiliated with the core phyla.

*Acidobacteria* was the bacterial phylum whose relative sequence abundances differed most between core and unique OTUs (Figure S5). It was the most abundant group of the core OTUs, with relative sequence abundances of 38.1±3.0% (surface) and 40.3±1.4% (subsurface). The relative abundance of the *Acidobacteria*, however, declined to 15.3±2.4% (surface) and 29.4±14.7% (subsurface) among the rare OTUs to as low as 4.6±2.7% (surface) and 6.5±6.8% among the unique OTUs. This decline in relative abundance is remarkable, in particular when compared to the relative sequence abundances of the *Proteobacteria*: 30.5±2.5% (surface) and 32.7±2.1% (subsurface) among core OTUs; 33.5±2.9% (surface) and 34.5±7.7% (subsurface) among rare OTUs; and 21.9±3.4% (surface) and 26.9±3.8% (subsurface) among unique OTUs. This discrepancy between *Acidobacteria* and *Proteobacteria* in their relative sequence abundances among unique OTUs possibly can be explained by the fact that all peat-inhabiting *Acidobacteria* are more or less uniform with regard to their lifestyles (i.e., acidophilic, aerobic or anaerobic chemoorganotrophs with weak hydrolytic capabilities) and therefore display the same good adaptation to the environmental conditions prevailing in acidic peat bogs. In contrast, proteobacteria exhibit many different lifestyles. Distinct taxonomic patterns between core and unique OTUs were also observed for the intraphylum comparison of proteobacterial OTUs, at both class and order levels (Figure S6).

Notably, the proportion of sequences that could not be affiliated with any bacterial group strongly increased from core to unique OTUs: 0.49±0.2% (surface) and 1.3±0.2% (subsurface) among the core OTUs; 6.4±0.5% (surface) and 6.1±1.9% (subsurface) among the rare OTUs; and 32.9±6.0% (surface) and 24.6±2.7% (subsurface) among the unique OTUs. These unassigned sequences may represent either novel bacterial groups or, despite denoising, artificial singletons and doubletons derived from abundant OTUs. The significantly lower proportion of acidobacterial sequences among the unique OTUs relative to the core OTUs, however, suggests that most of the denoised singletons and doubletons are valid and provide reliable taxonomic information. This conclusion is further corroborated by comparing, for denoised and non-denoised *Methylocystaceae*-like sequences, the putative sequencing error rates in two conserved 16****S rRNA gene regions. These rates were four to five times higher in the non-denoised sequences of *Methylocystaceae* singleton OTUs than in the denoised sequences of the two major *Methylocystaceae* OTUs. Given that no *Methylocystaceae* singleton OTUs were detected in the denoised sequence data sets, the *Methylocystaceae* singleton OTUs in the non-denoised sequence data sets were, most likely, of artificial origin and eliminated by denoising (Figure S7).

### Abundant OTUs in Surface and Subsurface Peat

**Figure 4:**
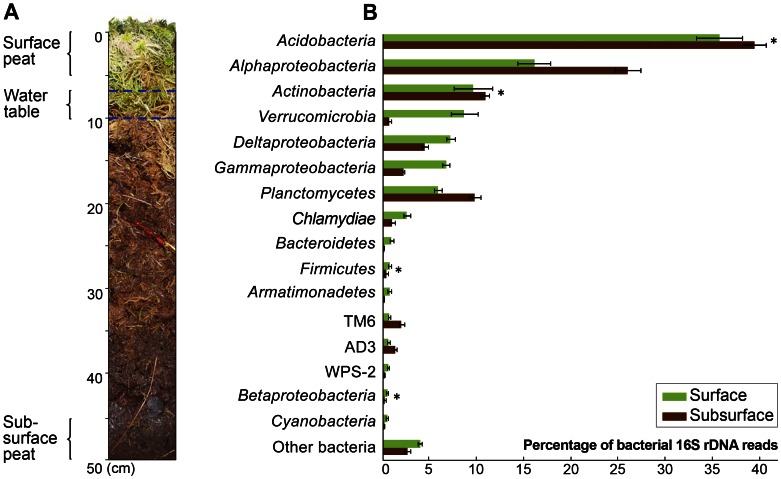
Bacterial community composition in surface (green bars) and subsurface (brown bars) peat. (A) Depth profile of *Sphagnum*-dominated peat bog. (B) Major taxa detected with a relative sequence abundance ≥0.5% are displayed. Column “other bacteria” indicates the combined relative sequence abundance of all the rare phyla and candidate divisions [each <0.5%] and of the taxonomically unclassified sequences. The error bars indicate the standard deviation of relative sequence abundances between the four subsamples. The number of 454 reads assigned to a particular taxon was significantly different between surface and subsurface peat based on two-tailed t-test (*P*<0.05), except for those four taxa indicated by asterisk.

Samples from both surface and subsurface peat were dominated by representatives of the *Acidobacteria* (35.5±2.4% and 39.2±1.2% of all classified sequences, respectively) and *Alphaproteobacteria* (15.9±1.7% and 25.8±1.4%) (Figure 4). The predominance of these groups had been reported in all previous studies of bacterial diversity in ombrotrophic peatlands of different geographic locations [16], [20]–[22], [24], [26]. Other major groups in surface and subsurface peat were the *Actinobacteria* (9.5±2.0% and 10.7±0.5% of all classified sequences, respectively), *Verrucomicrobia* (8.5±1.4% and 0.6±0.2%), *Planctomycetes* (5.8±0.4% and 9.7±0.6%), *Deltaproteobacteria* (7.1±0.4% and 4.4%±0.3%), and *Gammaproteobacteria* (6.6±0.4% and 2.1±0.1%).

**Figure 5:**
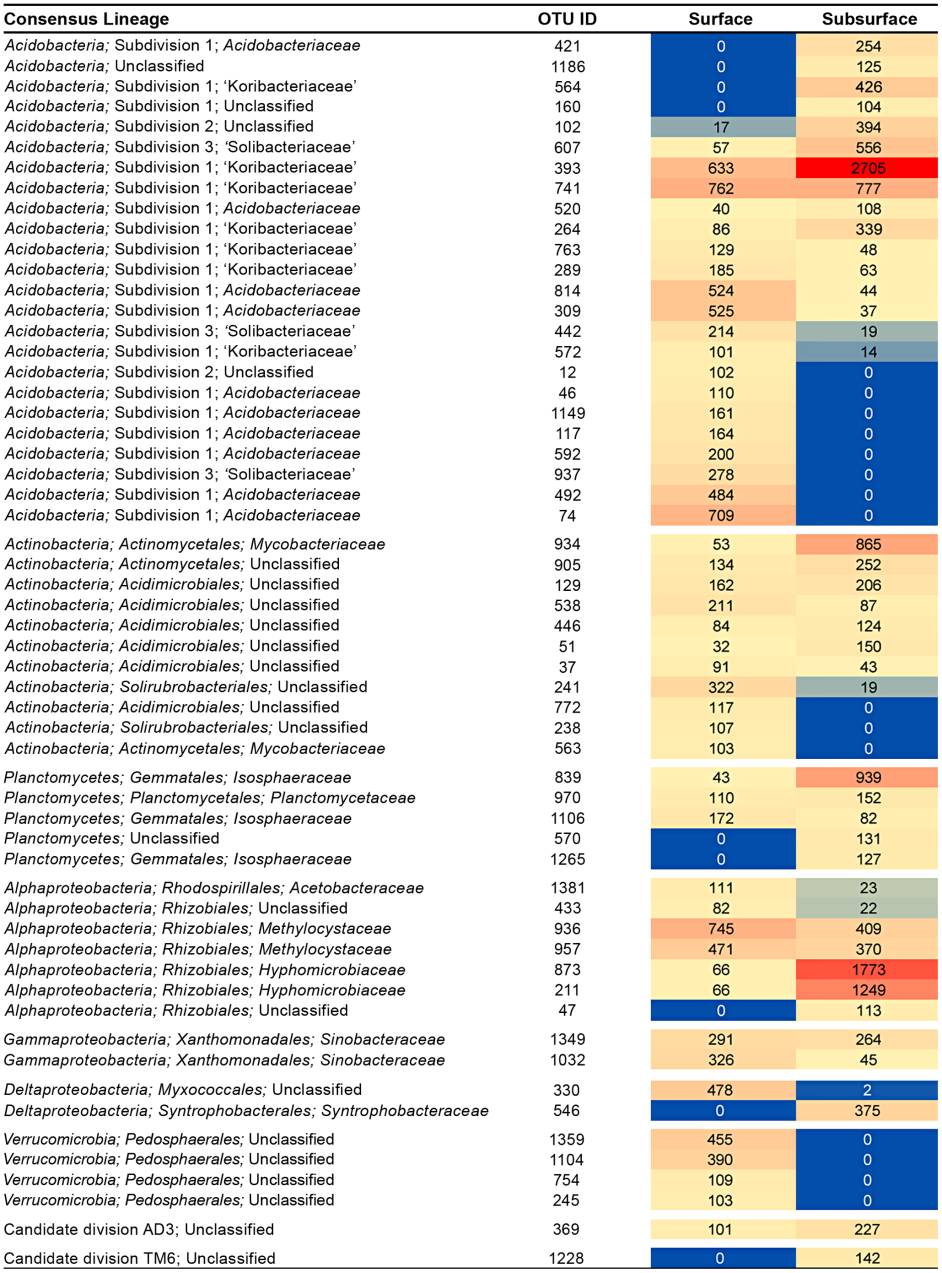
Heat map showing the most abundant OTUs and their distribution in surface and subsurface peat. OTUs are defined at a 97% sequence identity threshold. The digits indicate the total number of 454 reads recovered for the particular OTU from either surface (oxic) or subsurface (anoxic) peat. Low and high sequence abundances are highlighted in blue and red, respectively.

The most abundant OTUs were detected with similar relative sequence abundances in each of the four subsamples taken from either surface or subsurface peat. Their overall distribution in the two peat layers is displayed in Figure 5. The bacterial phylotypes representing these species-level OTUs were affiliated with subdivisions 1 and 3 of the *Acidobacteria*, the order *Rhizobiales* (families *Methylocystaceae* and *Hyphomicrobiaceae*) of the *Alphaproteobacteria*, the *Myxococcales* and *Syntrophobacterales* of the *Deltaproteobacteria,* the *Xanthomonadales* of the *Gammaproteobacteria*, the *Actinomycetales, Solirubrobacteriales*, and *Acidimicrobiales* of the *Actinobacteria,* the *Opitutales* and *Pedosphaerales* of the *Verrucomicrobia*, and the family *Isosphaeraceae* of the *Planctomycetes*. In most cases, these phylotypes were highly prevalent in either surface or subsurface peat, in correspondence to their preference for growth in either oxic or anoxic conditions. For example, the abundant representatives of the *Myxococcales* and *Verrucomicrobia* were detected only in the oxic peat, while those of the *Syntrophobacterales* and the candidate division TM6 were found exclusively in the anoxic zone of the peat profile (Figure 5). Some groups of bacteria, however, were detected with similar sequence abundances in the oxic and anoxic zones of the peat profile; among those members of the subdivision 1 *Acidobacteria, Actinobacteria*, *Alphaproteobacteria*, *Gammaproteobacteria*, and candidate division AD3. In particular, type II methanotrophs of the family *Methylocystaceae* were detected in both oxic and anoxic peat. These are known to have the ability to tolerate long periods of oxygen starvation and also to thrive in anoxic conditions by fermenting their characteristic storage compound, poly-β-hydroxybutyrate [45]. By contrast, type I methanotrophs of the family *Methylococcaceae* were detected only in the oxic surface layer. The ability to survive long periods of oxygen depletion has never been demonstrated for these type I methanotrophs, thereby confirming that all the samples taken from subsurface peat were consistently anoxic.

**Figure 6:**
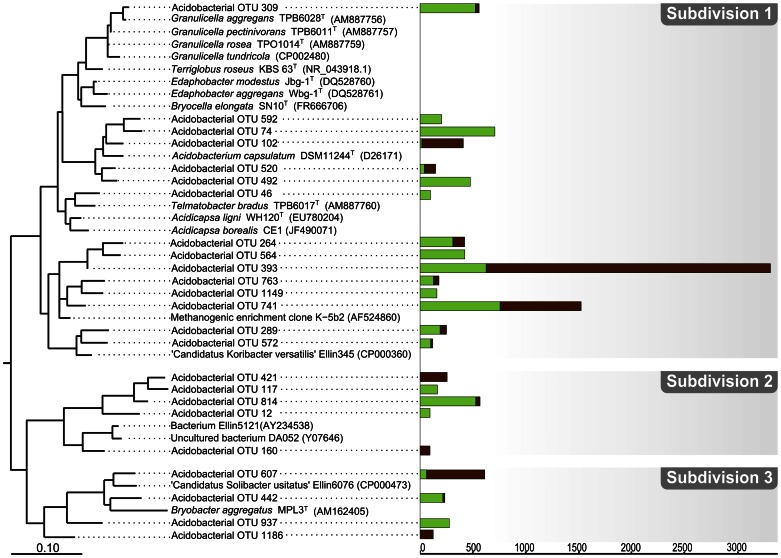
Phylogenetic tree showing the positions of peat bog 16 S rRNA gene sequences within the phylum ***Acidobacteria*** **.** Peat-derived reads are displayed in relation to 16****S rRNA gene sequences from cultured representatives of subdivisions 1 and 3 of the *Acidobacteria* and from other environmental samples. Only OTUs represented by at least 100 reads in one of the two sequence data sets are shown (compare with Figure 5). 16****S rRNA gene sequences from *Geothrix fermentans* H-5^T^ (U41563) and *Holophaga foetida* TMBS4^T^ (X77215), both being members of the acidobacterial subdivision 8, were used as an outgroup (not shown). Green and brown bars indicate the number of 454 reads retrieved for that branch from surface and subsurface peat, respectively. The distance bar indicates 0.1 substitutions per nucleotide position.

**Figure 7:**
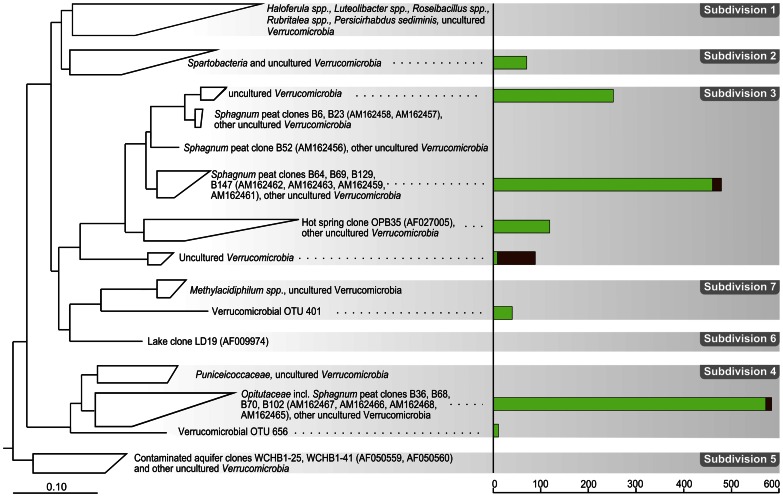
Phylogenetic tree showing the positions of peat bog 16 S rRNA gene sequences within the phylum ***Verrucomicrobia*** **.** Peat-derived reads are displayed in relation to 16****S rRNA gene sequences from cultivated representatives of subdivisions 1–7 of the *Verrucomicrobia* and from other environmental samples. Only OTUs represented by more than 10 reads in at least one of the two sequence data sets are shown. 16****S rRNA gene sequences from *Gemmata obscuriglobus* and five *Gemmata*-like planctomycetes (ABGO01000192, X81957, AF239694, AF239696, AF239697, AF239698) were used as an outgroup (not shown). Green and brown bars indicate the number of 454 reads retrieved for that branch from surface and subsurface peat, respectively. The distance bar indicates 0.1 substitutions per nucleotide position.

**Figure 8:**
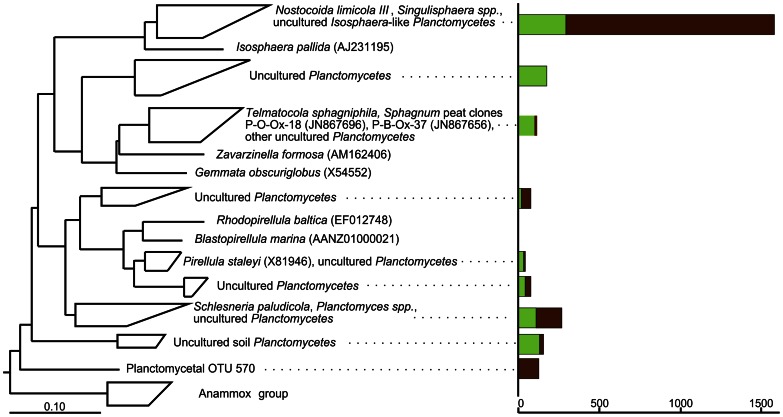
Phylogenetic tree showing the positions of 16 S rRNA gene sequences within the phylum ***Planctomycetes*** **.** Peat-derived reads are displayed in relation to 16****S rRNA gene sequences from cultivated representatives of the *Planctomycetes* and from other environmental samples. Only OTUs represented by more than 10 reads in at least one of the two sequence data sets are shown. 16****S rRNA gene sequences from *Geothrix fermentans* H-5^T^ (U41563) and *Holophaga foetida* TMBS4^T^ (X77215) were used as an outgroup (not shown). Green and brown bars indicate the number of 454 reads retrieved for the respective branch from surface and subsurface peat, respectively. The distance bar indicates 0.1 substitutions per nucleotide position.

**Figure 9:**
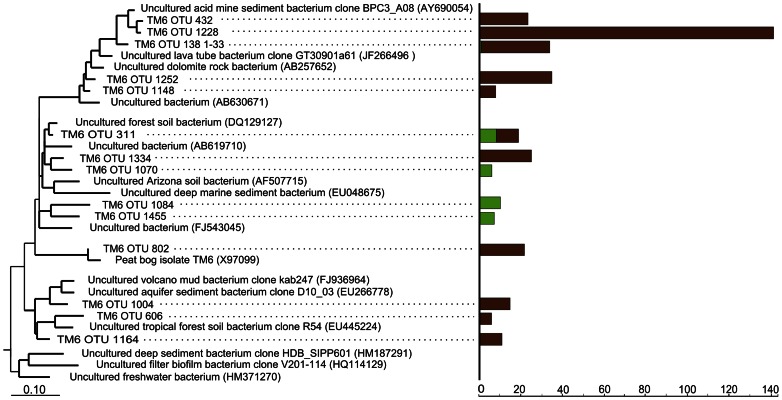
Phylogenetic tree showing the positions of 16 S rRNA gene sequences within the candidate division TM6. Peat-derived reads are displayed in relation to 16****S rRNA gene sequences from diverse environments. Only OTUs represented by more than 5 reads in at least one of the two sequence data sets are shown. 16****S rRNA gene sequences from *Geothrix fermentans* H-5^T^ (U41563) and *Holophaga foetida* TMBS4^T^ (X77215), both being members of the acidobacterial subdivision 8, were used as an outgroup (not shown). Green and brown bars indicate the total number of 454 reads retrieved for the respective branch from surface and subsurface peat, respectively. The distance bar indicates 0.1 substitutions per nucleotide position.

In summary, the most striking phylum-level differences in relative sequence abundance between surface and subsurface peat layers were detected for the *Verrucomicrobia*, but most of the other phyla also showed significant differences. Exceptions were *Acidobacteria*, *Actinobacteria*, *Firmicutes* and, at the subphylum level, *Betaproteobacteria* (Figure 4). In the following, the focus will be on the *Acidobacteria*, *Verrucomicrobia*, *Planctomycetes* and candidate division TM6; all of them representing poorly understood or elusive groups of bacteria that, however, are abundant in *Sphagnum*-dominated peat bogs. Their intraphylum diversity patterns differed between surface and subsurface peat (Figures 5 , 6 , 7 , 8 , 9).

### Acidobacteria

The largest groups of 16****S rRNA gene sequences in both surface and subsurface peat (6,710 and 7,141 reads, respectively) were affiliated with the *Acidobacteria*, providing further evidence that members of this phylum are the main component of bacterial communities in peatlands. The number of species-level OTUs was detected to be 105 and 69 for surface and subsurface peat, respectively. Of these, 21 OTUs were common to both sequence data sets. Taxonomy-based analysis classified most of these OTUs as belonging to subdivisions 1, 2, and 3 of the *Acidobacteria* (Table 1), while only a minor portion of the acidobacterial sequences (∼0.2–0.6%) was affiliated with subdivision 8. As mentioned above, the relative abundances of acidobacterial sequences were not significantly different between surface and subsurface peat (Figure 4). In contrast, the proportion of 16****S rRNA gene sequences, which were assigned to taxonomically characterized members of the *Acidobacteria*, was significantly higher in the surface layer than in the subsurface peat (14.8% versus 1.1% of all acidobacterial sequences). Apparently, there is a major lack of knowledge about acidobacteria that thrive in anoxic environments.

**Table 1:** Taxonomic assignment of 16****S rRNA gene sequences affiliated with the *Acidobacteria*, which were retrieved from surface and subsurface peat layers (the analysis was made in MOTHUR by using SILVA reference database, at a confidence threshold of 80%).

Subdivision	Genus	Surface layer		Subsurface layer	
		Percentage	No. of reads	Percentage	No. of reads
Subdivision 1	*Acidicapsa*	0.36	24	0	0
Subdivision 1	*Acidobacterium*	1.3	86	0	0
Subdivision 1	*Edaphobacter*	0.12	8	0.59	37
Subdivision 1	*Granulicella*	7.3	482	0	0
Subdivision 1	Uncharacterized	68.83	4547	89.46	5583
Subdivision 2	Uncharacterized	2.72	180	4.34	271
Subdivision 3	*Candidatus* Solibacter	5.54	366	0.24	15
Subdivision 3	Uncharacterized	0.03	2	0	0
Subdivision 8	*Holophaga*	0.15	10	0.22	14
Subdivision 8	Uncharacterized	0.42	28	0.02	1
N/A	Unclassified *Acidobacteria*	13.22	873	5.13	320

The depth distribution of some acidobacterial phylotypes agreed well with the currently available information on the lifestyle of closely related organisms (Figure 6). For example, *Granulicella*- and *Bryobacter*-related phylotypes (OTUs 309 and 442) were found mostly in the oxic peat, which coincides with the fact that members of these two genera are obligate aerobes [46]–[48]. Another example may be the detection of the acidobacterial OTUs 393 and 741 primarily in the anoxic peat. These phylotypes were most closely related to a 16****S rRNA gene clone sequence from a methanogenic consortium enriched from the Siberian peat bog Bakchar [49]. Several phylotypes within subdivisions 2 and 3 (OTUs 160, 421, and 1186) were found exclusively in the subsurface peat. These examples suggest that the depth distribution of the acidobacterial phylotypes may adequately reflect their preferences for either oxic or anoxic conditions and that a considerable proportion of as-yet-uncultivated subdivision 1 and 3 *Acidobacteria* may have anaerobic (either facultative or obligate) lifestyles. Most cultivated members of these two subdivisions were originally characterized to be obligate aerobes. The recent description of the facultative anaerobe *Telmatobacter bradus* and documentation of a fermentative metabolism in *Acidobacterium capsulatum* [27], however, demonstrate that our former view of subdivision 1 *Acidobacteria* as obligately aerobic organisms might have been incorrect. Notably, the most abundant bacterial phylotype detected in our study (represented by the OTU 393) was affiliated with subdivision 1 *Acidobacteria* and displayed a clear preference for anoxic peat (Figures 5 and 6).

### Verrucomicrobia

Members of the *Verrucomicrobia* exhibited a clear preference for the oxic surface layer (Figure 4). In total, this phylum was represented by 1,731 reads of which most were retrieved from the surface peat (1,611 reads corresponding to 40 OTUs) and affiliated with subdivisions 2, 3, and 4 of the *Verrucomicrobia* (Figure 7). Of these, subdivisions 3 and 4 were most frequently detected in the 454 libraries. The numerically predominant group of 16****S rRNA gene sequences formed a coherent cluster with *Opitutus terrae*, a strictly anaerobic polysaccharide-utilizing ultramicrobacterium [50]. Members of this lineage were previously detected in an anoxic rice paddy soil [51] and at the oxic-anoxic interface in the Siberian peat bog Bakchar ([21]; clones B36, B68, B70, and B102; accession numbers AM162467, AM162466, AM162468, and AM162465). The results of our study, however, suggest that this lineage within the *Verrucomicrobia* contains both aerobic and anaerobic organisms. Another large group of sequences belonged to a broad phylogenetic cluster within *Verrucomicrobia* subdivision 3, for which cultured representatives have not yet been reported (Figure 7). These sequences also clustered together with 16****S rRNA gene clones retrieved from the peat bog Bakchar (clones B6, B23, B52, B69, B129, and B147; accession numbers AM162458, AM162457, AM162456, AM162463, AM162459, and AM162461). This group within subdivision 3 appears to be highly characteristic of peat bog environments and, most likely, is represented by aerobic bacteria. Only a minor group of verrucomicrobial 16****S rRNA gene sequences (120 reads) was retrieved from the anoxic peat layer (Figure 7), while only two subdivision 3-related phylotypes (OTU 534, 26 reads; and OTU 914, 19 reads) and one subdivision 4-related phylotype (OTU 586, 13 reads) were detected exclusively in the subsurface peat.

We did not find any firm evidence for the presence of *Methylacidiphilum*-like methanotrophic *Verrucomicrobia* [31] in the acidic peat samples examined in our study. A group of forty 16****S rRNA gene sequences (OTU 401), which were retrieved exclusively from the surface peat, showed a distant relationship (87% sequence identity) to recognized verrucomicrobial methanotrophs and grouped next to the *Methylacidiphilaceae* (subdivision 7 *Verrucomicrobia*) in our treeing analysis (Figure 7). These sequences, however, displayed nearly the same level of identity (84%) to the 16****S rRNA gene sequence from subdivision 6 freshwater bacterium LD19 (accession number AF009974) and their exact phylogenetic affiliation could not be inferred. The occurrence of acidophilic verrucomicrobial methanotrophs in acidic wetlands, therefore, remains obscure.

### Planctomycetes

The number of species-level OTUs was detected to be 73 (1,091 reads) and 40 (1,805 reads) for surface and subsurface peat, respectively. Of these, only 5 OTUs were common to both sequence data sets. Most 16****S rRNA gene sequences representing this phylum in acidic peat belonged to the lineage defined by '*Nostocoida limicola*' III, members of the genus *Singulisphaera* and uncultivated *Isosphaera*-like planctomycetes (Figure 8). Despite the fact that all currently described members of this lineage are aerobes, most sequences affiliated with this lineage were retrieved from the anoxic peat layer. Two other groups of 16****S rRNA gene sequences represented *Telmatocola*- and *Schlesneria*/*Planctomyces*-like bacteria. The former group was more abundant at the peat surface, while the latter was equally abundant in the oxic and anoxic zones. This agrees well with our current knowledge of *Telmatocola* as an obligately aerobic bacterium and of *Schlesneria* as a facultative aerobe [28], [52]. One relatively large group of 16****S rRNA gene sequences found exclusively in the subsurface peat (OTU 570, 131 reads) represented a yet-uncultivated planctomycete lineage, which was equally divergent (≥15% 16****S rRNA gene sequence difference) from both the *Planctomycetales* and the anaerobic anammox planctomycetes.

### Candidate Division TM6

This poorly characterized bacterial group was less abundant but more diverse in the surface layer (115 reads corresponding to 46 species-level OTUs) than in the subsurface peat (358 reads corresponding to 34 OTUs). A total of only 6 OTUs were common to both sequence data sets, indicating that members of candidate division TM6 have a clear preference for either oxic or anoxic conditions. To date, no information about the physiology of these bacteria has been available. Our results, however, suggest that the abundant peat-inhabiting representatives of candidate division TM6 are obligate anaerobes (Figure 9). The first 16****S rRNA gene sequences representing this bacterial group were retrieved from an acidic (pH 2.7) peat, sampled at a depth of 40 cm. Sampling site was a peat bog near Gifhorn, Germany [19]. The sequences were named TM for ‘Torf, Mittlere Schicht’ ( =  peat, middle layer). Representatives of the TM6 group are commonly found in peatlands [26], but can also be detected in other environments. These include biofilms in water distribution systems [53], aquifer sediments [54], microbial mat communities in hypersaline lagoons [55], and other habitats.

In summary, the use of 454-pyrosequencing allowed us to elucidate the bacterial diversity in surface and subsurface layers of an acidic *Sphagnum*-dominated peat bog. Separate analysis of subsamples had no major effect on the taxonomic patterns of the abundant OTUs, while those of the rare and unique OTUs greatly varied among the subsamples. OTU richness was twice higher at the surface than in subsurface peat, while only a minor proportion of OTUs were common to both datasets. Apparently, the overall environmental conditions in *Sphagnum*-dominated peat bogs, such as high acidity, low temperature and low nutrient content, determined *Acidobacteria*, *Proteobacteria*, *Actinobacteria*, and *Planctomycetes* to be the dominant phylum-level groups in both the oxic surface layer and the anoxic zone. However, various members of these groups displayed a clear preference for either oxic or anoxic conditions. The depth distribution patterns of *Acidobacteria* and *Planctomycetes* suggest that many as-yet-uncultivated representatives of these two phyla have anaerobic lifestyles. However, anammox planctomycetes were not detected. Oxygen availability was of critical importance for the peat-inhibiting *Verrucomicrobia*, limiting their presence primarily to the oxic surface layer. In contrast, the abundant peat-inhabiting members of the candidate division TM6 appear to be obligate anaerobes, thereby providing novel insights into their physiological adaptations. Our data suggest that the bacterial communities in the oxic and anoxic zones of northern peatlands are highly diverse and taxonomically distinct, reflecting the different abiotic conditions along the bog profile.

## Supporting Information

Figure S1 **Principal coordinate analysis of bacterial community composition in surface and subsurface peat based on weighted UniFrac distance matrices.** The subsample sequence data sets from the surface layer (blue) were separated from those obtained from subsurface peat (red) by the first principal component, which explained 91% of variation. In contrast, the variations between the four subsample sequence data sets obtained from either the surface layer or the subsurface peat were described by the second and third principal components (at maximum 4% and 1.7%, respectively).
(TIF)

Figure S2 **Shannon diversity index of bacterial communities in surface and subsurface peat.** The curves show the relation between changes in the Shannon diversity index and the number of randomly sampled sequences, separately for each subsample. The curves for the subsamples from surface and subsurface peat are shown by solid and dashed lines, respectively. Color code is the same as used in Figure 1.
(TIF)

Figure S3 **Relative abundance of bacterial phyla and candidate divisions among rare OTUs.** Rare OTUs are those detected in two or three subsamples, but not in all four subsamples, of the respective peat layer. Phyla and candidate divisions marked by an asterisk were detected only in the surface layer, while those marked by a cross are unique to subsurface peat.
(TIF)

Figure S4 **Read number distribution among OTUs that were detected in only a single subsample (unique OTUs, red) or in two (green) or three (purple) subsamples (rare OTUs).** (A) Surface layer and (B) subsurface peat.
(TIF)

Figure S5 **Bacterial phyla and candidate divisions that exhibited significant differences in their relative sequence abundances between core OTUs (blue) and unique OTUs (yellow).** (A) Surface layer and (B) subsurface peat.
(TIF)

Figure S6 **Relative abundance of proteobacterial subgroups in surface and subsurface peat, separately analyzed for core and unique OTUs.** Analysis at (A) class level and (B) order level. Core OTUs are those detected in all four subsamples, while unique OTUs were detected in only a single subsample. Order-level groups marked by an asterisk were detected only in surface peat, while those marked by a cross are unique to subsurface peat.
(TIF)

Figure S7 **Putative sequencing errors in denoised versus non-denoised sequence data.** The putative error rates (A) were calculated for two conserved regions – “Site 1″ (GTGGAGCATGTGGTTTAATTCGAAGCAACGCG; B, D) and “Site 2″ (TGGCTGTCGTCAGCTCGTGTC; C, E), using a set of 798 denoised *Methylocystaceae* sequences (B, C) and a set of 14 *Methylocystaceae* singleton sequences obtained when denoising was not applied (D, E). Note that *Methylocystaceae* singleton OTUs were not found in the denoised sequence data sets.
(TIF)

Table S1 **Taxonomic assignment of 16 S rRNA gene sequences affiliated with the** ***Archaea*** **, which were retrieved from surface and subsurface peat layers (the analysis was made in MOTHUR by using SILVA reference database, at a confidence threshold of 80%).**
(DOCX)

Table S2 **Taxonomic assignment of 18 S rRNA gene sequences affiliated with the** ***Eukarya*** **, which were retrieved from surface and subsurface peat layers (the analysis was made in MOTHUR by using SILVA reference database, at a confidence threshold of 80%).**
(DOCX)
