# Supplementary figures and images for: Pyrosequencing-Based Assessment of the Bacteria Diversity in Surface and Subsurface Peat Layers of a Northern Wetland, with Focus on Poorly Studied Phyla and Candidate Divisions
Source: PLoS One. 2013 May 21;8(5):e63994. doi: 10.1371/journal.pone.0063994 (PMC3660313; doi:10.1371/journal.pone.0063994)

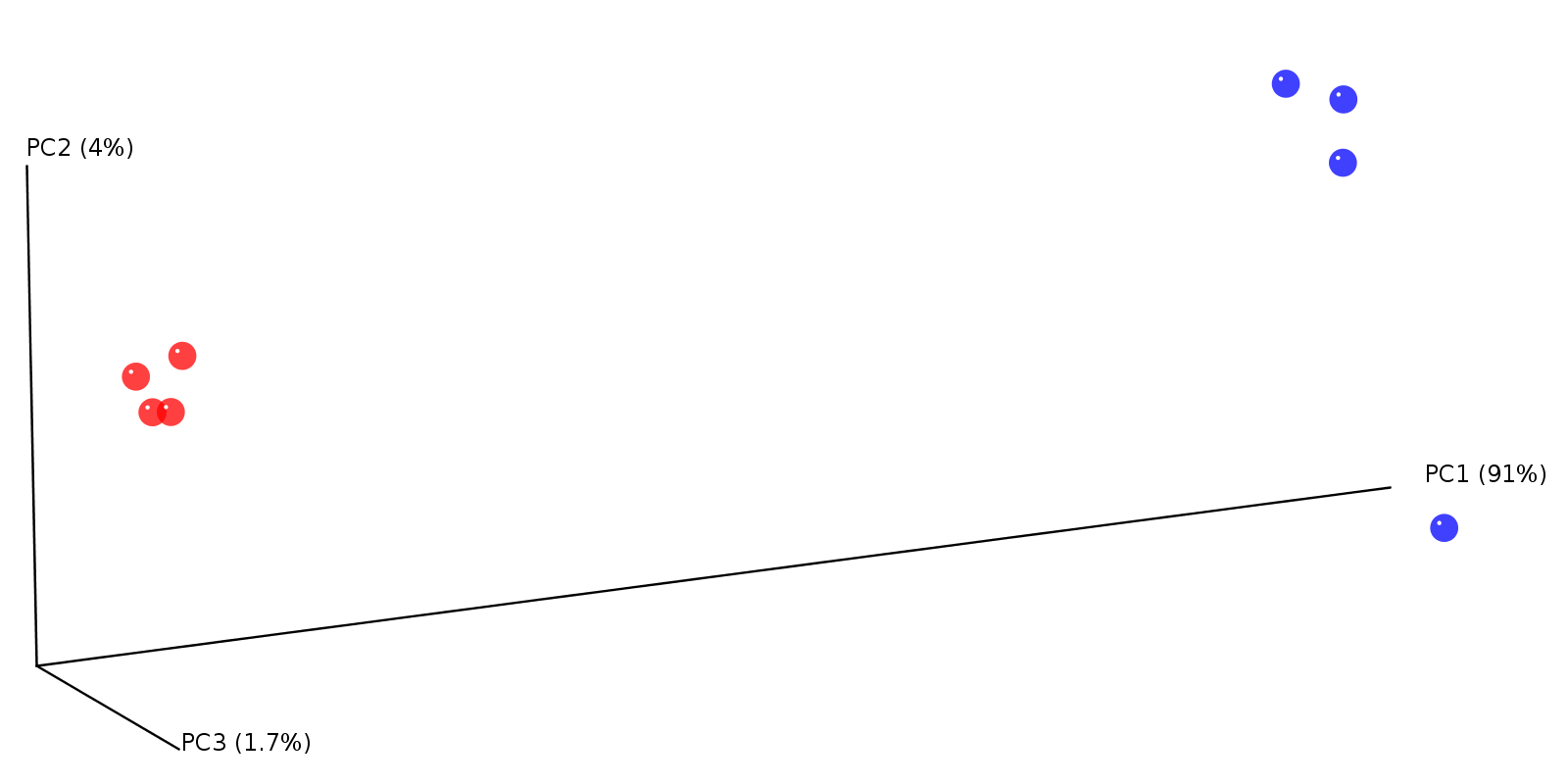

Supplement: Figure S1 — Principal coordinate analysis of bacterial community composition in surface and subsurface peat based on weighted UniFrac distance matrices. The subsample sequence data sets from the surface layer (blue) were separated from those obtained from subsurface peat (red) by the first principal component, which explained 91% of variation. In contrast, the variations between the four subsample sequence data sets obtained from either the surface layer or the subsurface peat were described by the second and third principal components (at maximum 4% and 1.7%, respectively). (TIF) [file pone.0063994.s001.tif]

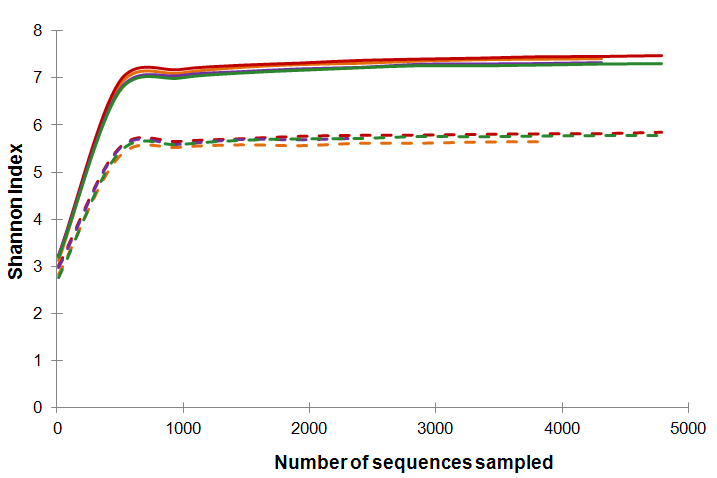

Supplement: Figure S2 — Shannon diversity index of bacterial communities in surface and subsurface peat. The curves show the relation between changes in the Shannon diversity index and the number of randomly sampled sequences, separately for each subsample. The curves for the subsamples from surface and subsurface peat are shown by solid and dashed lines, respectively. Color code is the same as used in Figure 1 . (TIF) [file pone.0063994.s002.tif]

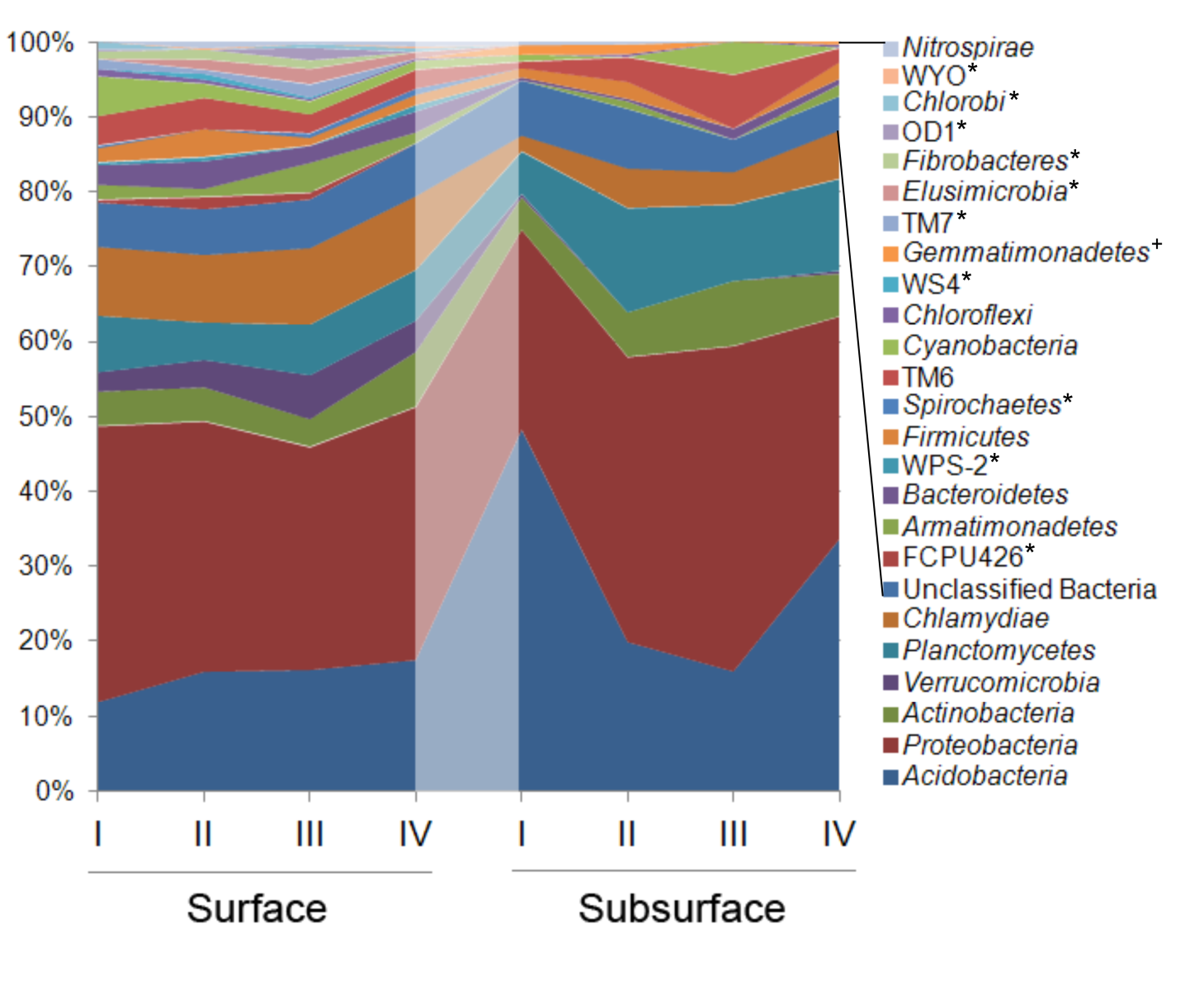

Supplement: Figure S3 — Relative abundance of bacterial phyla and candidate divisions among rare OTUs. Rare OTUs are those detected in two or three subsamples, but not in all four subsamples, of the respective peat layer. Phyla and candidate divisions marked by an asterisk were detected only in the surface layer, while those marked by a cross are unique to subsurface peat. (TIF) [file pone.0063994.s003.tif]

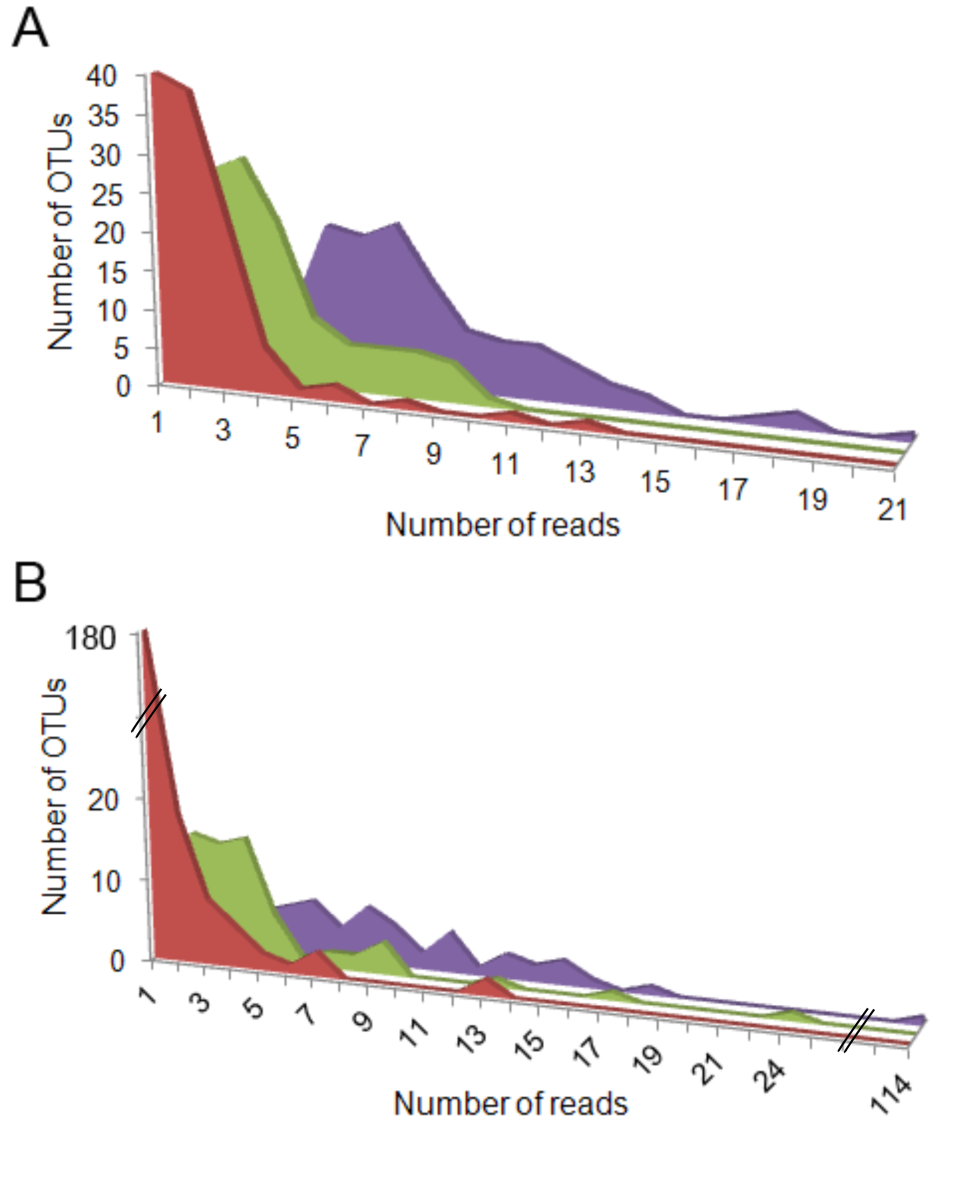

Supplement: Figure S4 — Read number distribution among OTUs that were detected in only a single subsample (unique OTUs, red) or in two (green) or three (purple) subsamples (rare OTUs). (A) Surface layer and (B) subsurface peat. (TIF) [file pone.0063994.s004.tif]

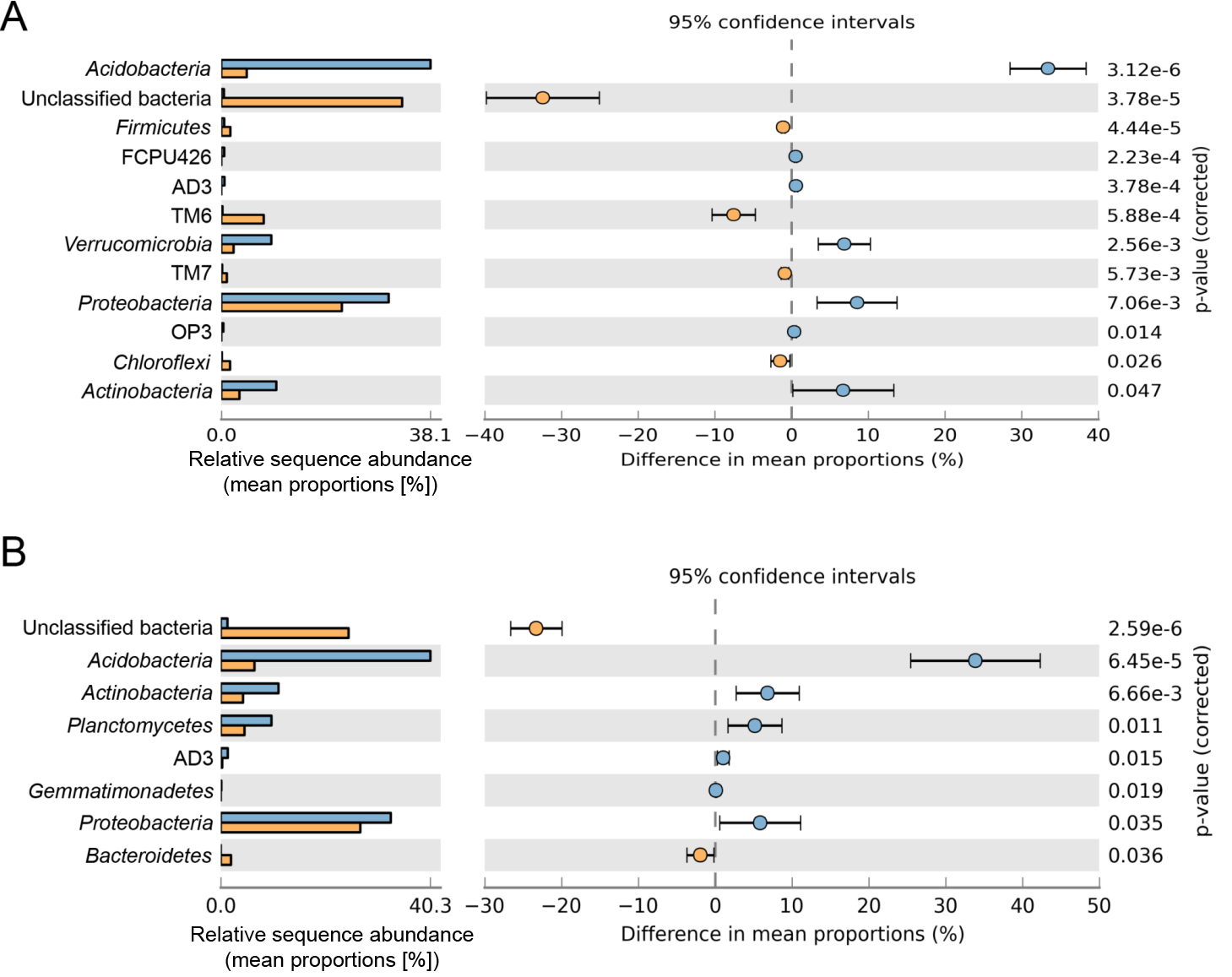

Supplement: Figure S5 — Bacterial phyla and candidate divisions that exhibited significant differences in their relative sequence abundances between core OTUs (blue) and unique OTUs (yellow). (A) Surface layer and (B) subsurface peat. (TIF) [file pone.0063994.s005.tif]

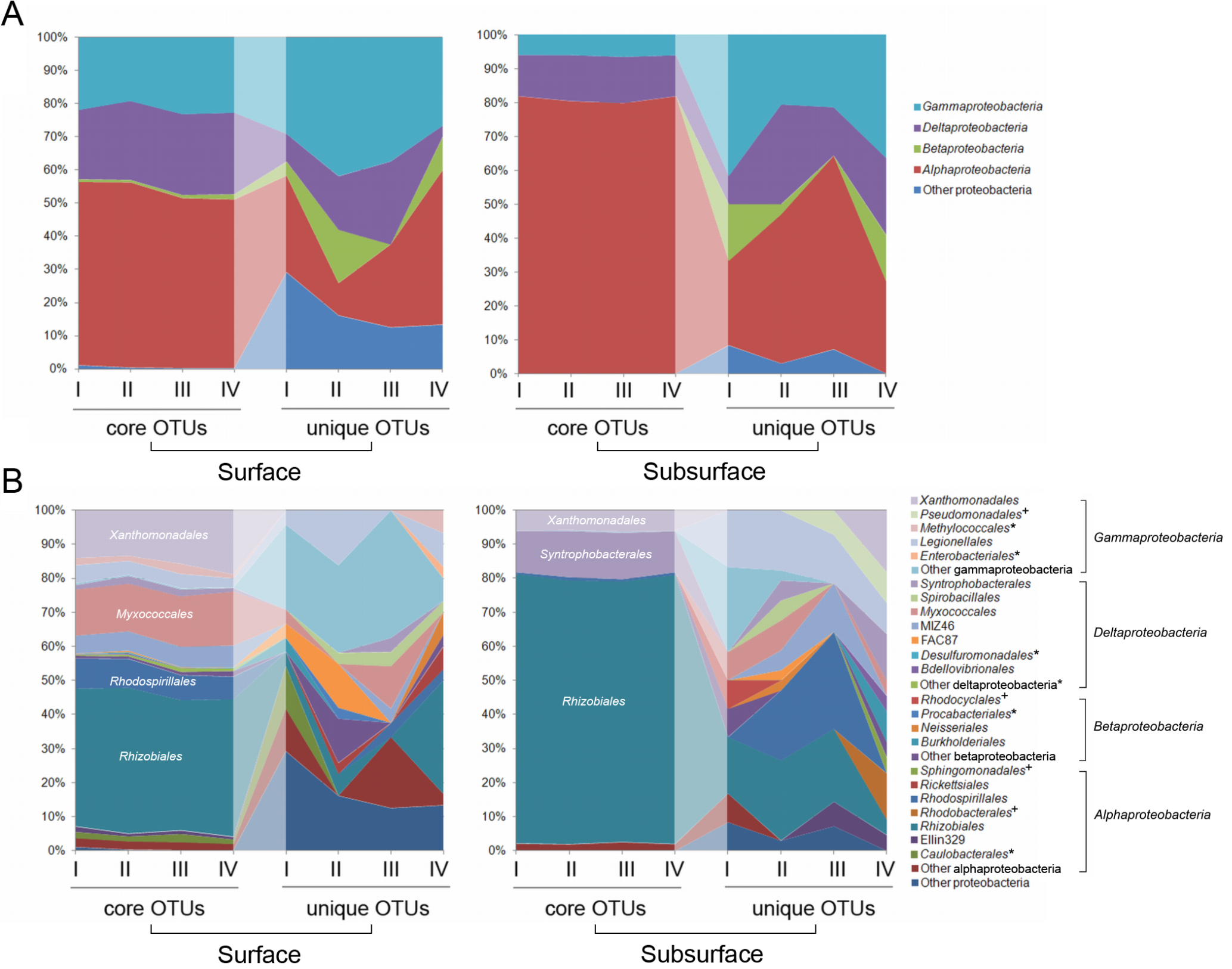

Supplement: Figure S6 — Relative abundance of proteobacterial subgroups in surface and subsurface peat, separately analyzed for core and unique OTUs. Analysis at (A) class level and (B) order level. Core OTUs are those detected in all four subsamples, while unique OTUs were detected in only a single subsample. Order-level groups marked by an asterisk were detected only in surface peat, while those marked by a cross are unique to subsurface peat. (TIF) [file pone.0063994.s006.tif]

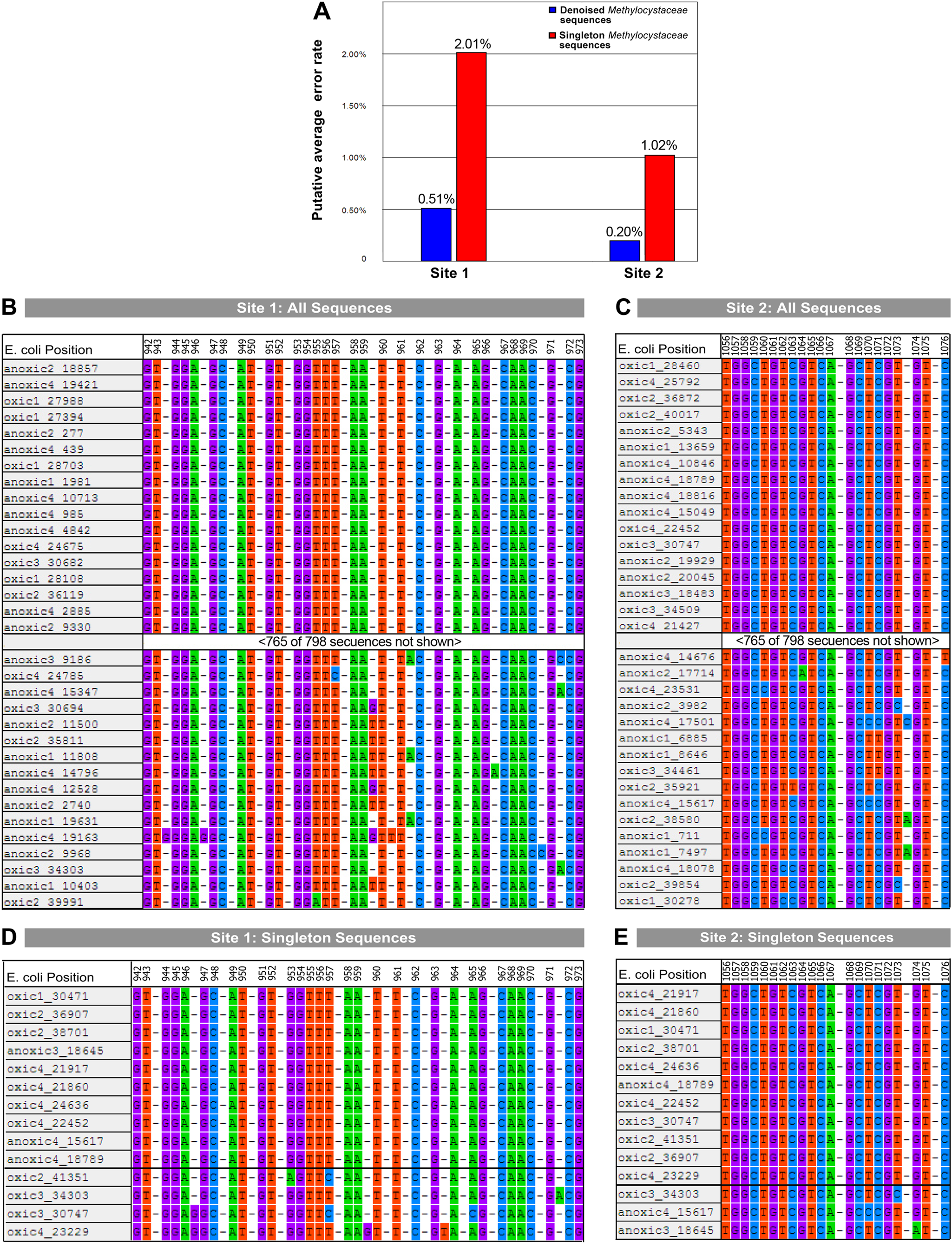

Supplement: Figure S7 — Putative sequencing errors in denoised versus non-denoised sequence data. The putative error rates (A) were calculated for two conserved regions – “Site 1″ (GTGGAGCATGTGGTTTAATTCGAAGCAACGCG; B, D) and “Site 2″ (TGGCTGTCGTCAGCTCGTGTC; C, E), using a set of 798 denoised Methylocystaceae sequences (B, C) and a set of 14 Methylocystaceae singleton sequences obtained when denoising was not applied (D, E). Note that Methylocystaceae singleton OTUs were not found in the denoised sequence data sets. (TIF) [file pone.0063994.s007.tif]
