# Supplementary material for: Pyrosequencing-Based Assessment of the Bacteria Diversity in Surface and Subsurface Peat Layers of a Northern Wetland, with Focus on Poorly Studied Phyla and Candidate Divisions
Source: PLoS One. 2013 May 21;8(5):e63994. doi: 10.1371/journal.pone.0063994 (PMC3660313; doi:10.1371/journal.pone.0063994)
Supplement: Table S1 — Taxonomic assignment of 16 S rRNA gene sequences affiliated with the Archaea , which were retrieved from surface and subsurface peat layers (the analysis was made in MOTHUR by using SILVA reference database, at a confidence threshold of 80%). (DOCX) [file pone.0063994.s008.docx]

**Table S1.** Taxonomic assignment of 16S rRNA gene sequences affiliated with the *Archaea*, which were retrieved from surface and subsurface peat layers (the analysis was made in MOTHUR by using SILVA reference database, at a confidence threshold of 80%).

|  | **Surface layer** | | **Subsurface layer** | |
| --- | --- | --- | --- | --- |
|  | Percentage | No. of reads | Percentage | No. of reads |
| ***Crenarchaeota*** |  |  |  |  |
| Uncultured *Crenarchaeota* | 13.79 | 4 | 0.95 | 20 |
| ***Euryarchaeota*** |  |  |  |  |
| *Methanobacteria* |  |  |  |  |
| *Methanobacterium* | 3.45 | 1 | 0.14 | 3 |
| *Methanomicrobia* |  |  |  |  |
| *Methanocella* | 3.45 | 1 | 0.19 | 4 |
| *Methanosarcina* | 6.90 | 2 | 0.76 | 16 |
| Candidatus Methanoregula | 58.62 | 17 | 0.38 | 8 |
| Uncultured *Methanomicrobia* | 3.45 | 1 | 71.27 | 1501 |
| *Thermoplasmata* |  |  |  |  |
| Uncultured *Thermoplasmatales* | 3.45 | 1 | 6.13 | 129 |
| **Uncultured Archaea** | 6.90 | 2 | 20.18 | 425 |
| **Total:** |  | **29** |  | **2106** |
