# Supplementary material for: Pyrosequencing-Based Assessment of the Bacteria Diversity in Surface and Subsurface Peat Layers of a Northern Wetland, with Focus on Poorly Studied Phyla and Candidate Divisions
Source: PLoS One. 2013 May 21;8(5):e63994. doi: 10.1371/journal.pone.0063994 (PMC3660313; doi:10.1371/journal.pone.0063994)
Supplement: Table S2 — Taxonomic assignment of 18 S rRNA gene sequences affiliated with the Eukarya , which were retrieved from surface and subsurface peat layers (the analysis was made in MOTHUR by using SILVA reference database, at a confidence threshold of 80%). (DOCX) [file pone.0063994.s009.docx]

**Table S2.** Taxonomic assignment of 18S rRNA gene sequences affiliated with the *Eukarya*, which were retrieved from surface and subsurface peat layers (the analysis was made in MOTHUR by using SILVA reference database, at a confidence threshold of 80%).

|  | **Surface layer** | | **Subsurface layer** | |
| --- | --- | --- | --- | --- |
|  | Percentage | No. of reads | Percentage | No. of reads |
| ***Fungi*** |  |  |  |  |
| *Ascomycota* |  |  |  |  |
| *Saccharomycetes* | 0.19 | 5 | 1.90 | 2 |
| *Sordariomycetes* | 0.95 | 25 | 0 | 0 |
| Unclassified *Ascomycota* | 1.33 | 35 | 0 | 0 |
| *Basidiomycota* |  |  |  |  |
| *Agaricomycetes* | 2.97 | 78 | 0 | 0 |
| *Microbotryomycetes* | 0.34 | 9 | 7.62 | 8 |
| *Exobasidiomycetes* | 0 | 0 | 12.38 | 13 |
| Unclassified *Fungi* | 0.99 | 26 | 0 | 0 |
| ***Alveolata*** |  |  |  |  |
| *Apicomplexa* | 0.04 | 1 | 0 | 0 |
| *Ciliophora* | 0.84 | 22 | 0.95 | 1 |
| *Dinophyceae* | 0 | 0 | 7.62 | 8 |
| Unclassified *Alveolata* | 0.34 | 9 | 0 | 0 |
| ***Metazoa*** |  |  |  |  |
| *Annelida* |  |  |  |  |
| *Oligochaeta* | 22.50 | 591 | 0 | 0 |
| *Arachnida* | 8.53 | 224 | 0 | 0 |
| *Ascaridida* | 2.44 | 64 | 0 | 0 |
| *Platyhelminthes* |  |  |  |  |
| *Catenulida* | 4.57 | 120 | 0 | 0 |
| *Rhabdocoela* | 1.03 | 27 | 0 | 0 |
| *Chordata* |  |  |  |  |
| *Vertebrata* | 0 | 0 | 17.14 | 18 |
| Unclassified *Metazoa* | 22.15 | 582 | 6.67 | 7 |
| ***Viridiplantae*** |  |  |  |  |
| *Chlorophyta* |  |  |  |  |
| Unclassified *Chlorophyceae* | 0.15 | 4 | 2.86 | 3 |
| *Streptophyta* |  |  |  |  |
| *Bryophyta* | 0.72 | 19 | 0 | 0 |
| *Tracheophyta* | 0.84 | 22 | 0.95 | 1 |
| Unclassified *Viridiplantae* | 1.79 | 47 | 0 | 0 |
| **Stramenopiles** |  |  |  |  |
| *Mallomonas* | 0.19 | 5 | 0 | 0 |
| Unclassified Stramenopiles | 0.61 | 16 | 0 | 0 |
| **Unclassified *Eukaryota*** | 26.49 | 696 | 41.90 | 44 |
| **Total:** |  | **2627** |  | **105** |
